# Ferrihydrite-mediated methanotrophic nitrogen fixation in paddy soil under hypoxia

**DOI:** 10.1093/ismeco/ycae030

**Published:** 2024-03-04

**Authors:** Linpeng Yu, Rong Jia, Shiqi Liu, Shuan Li, Sining Zhong, Guohong Liu, Raymond Jianxiong Zeng, Christopher Rensing, Shungui Zhou

**Affiliations:** Fujian Provincial Key Laboratory of Soil Environmental Health and Regulation, College of Resources and Environment, Fujian Agriculture and Forestry University, Fuzhou 350002, China; Fujian Provincial Key Laboratory of Soil Environmental Health and Regulation, College of Resources and Environment, Fujian Agriculture and Forestry University, Fuzhou 350002, China; Key Laboratory of Land Resources Evaluation and Monitoring in Southwest China, Ministry of Education, Sichuan Normal University, Chengdu, Sichuan Province 610066, China; Fujian Provincial Key Laboratory of Soil Environmental Health and Regulation, College of Resources and Environment, Fujian Agriculture and Forestry University, Fuzhou 350002, China; Fujian Provincial Key Laboratory of Soil Environmental Health and Regulation, College of Resources and Environment, Fujian Agriculture and Forestry University, Fuzhou 350002, China; Fujian Provincial Key Laboratory of Soil Environmental Health and Regulation, College of Resources and Environment, Fujian Agriculture and Forestry University, Fuzhou 350002, China; Agricultural Bio-resources Research Institute, Fujian Academy of Agricultural Sciences, Fuzhou 350003, China; Fujian Provincial Key Laboratory of Soil Environmental Health and Regulation, College of Resources and Environment, Fujian Agriculture and Forestry University, Fuzhou 350002, China; Fujian Provincial Key Laboratory of Soil Environmental Health and Regulation, College of Resources and Environment, Fujian Agriculture and Forestry University, Fuzhou 350002, China; Fujian Provincial Key Laboratory of Soil Environmental Health and Regulation, College of Resources and Environment, Fujian Agriculture and Forestry University, Fuzhou 350002, China

**Keywords:** methane oxidation, biological nitrogen fixation, methane-oxidizing bacteria, iron reduction, metagenomics

## Abstract

Biological nitrogen fixation (BNF) by methanotrophic bacteria has been shown to play an important role in maintaining fertility. However, this process is still limited to aerobic methane oxidation with sufficient oxygen. It has remained unknown whether and how methanotrophic BNF proceeds in hypoxic environments. Herein, we incubated paddy soils with a ferrihydrite-containing mineral salt medium to enrich methanotrophic bacteria in the presence of methane (20%, v/v) under oxygen constraints (0.27%, v/v). The resulting microcosms showed that ferrihydrite-dependent aerobic methane oxidation significantly contributed (81%) to total BNF, increasing the ^15^N fixation rate by 13-fold from 0.02 to 0.28 μmol ^15^N_2_ (g dry weight soil) ^-1^ d^−1^. BNF was reduced by 97% when ferrihydrite was omitted, demonstrating the involvement of ferrihydrite in methanotrophic BNF. DNA stable-isotope probing indicated that *Methylocystis*, *Methylophilaceae*, and *Methylomicrobium* were the dominant methanotrophs/methylotrophs that assimilated labeled isotopes (^13^C or ^15^N) into biomass. Metagenomic binning combined with electrochemical analysis suggested that *Methylocystis* and *Methylophilaceae* had the potential to perform methane-induced BNF and likely utilized riboflavin and *c*-type cytochromes as electron carriers for ferrihydrite reduction. It was concluded that ferrihydrite mediated methanotrophic BNF by methanotrophs/methylotrophs solely or in conjunction with iron-reducing bacteria. Overall, this study revealed a previously overlooked yet pronounced coupling of iron-dependent aerobic methane oxidation to BNF and improves our understanding of methanotrophic BNF in hypoxic zones.

## Graphical Abstract

**Graphical Abstract:**
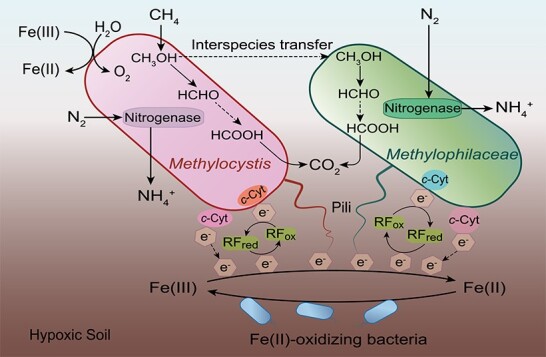

## Introduction

Industrial nitrogen fertilizer has widely been applied to enhance the nitrogen-limited primary productivity and pursue higher crop yields. However, excessive usage of nitrogen fertilizer and the low utilization efficiency inevitably give rise to a range of environmental hazards, including soil acidification, groundwater nitrate pollution, greenhouse gas (N_2_O) emission, and water eutrophication [1–4]. Apart from industrial nitrogen fertilizer, biological nitrogen fixation (BNF) also plays a non-negligible role in maintaining soil fertility and was estimated to provide >45 kg N ha^−1^ for unfertilized paddy soil annually [5]. Better insight into the mechanisms underlying BNF is of great significance when aiming to augment the soil BNF activity and reducing the usage of industrial nitrogen fertilizer and the environmental nitrogen burden.

The carbon and energy source of BNF in paddy soil generally come from soil organic matter or root exudates. The bioavailability of carbon and energy source often becomes a major limiting factor for BNF [6]. Methane is another potential, but often overlooked carbon and energy source for BNF in paddy soil, with methane being released in a large amount to the atmosphere via the diffusion, ebullition, or rice aerenchyma transport [7]. Methanotrophic BNF is not only able to function as a biofilter for the greenhouse gas methane but also supplies a large amount of nitrogen for plants. Boosting the methanotrophic BNF efficiency is beneficial for both agricultural sustainability and global climate balance. It was estimated that methanotrophic BNF is able to increase the N reserve by 30 kg N ha^−1^ over one rice growing season and contribute to 30% of the total nitrogen accumulation (i.e. 100 kg N ha^−1^) [8]. Methanotrophic BNF has also been shown to account for 65% of total nitrogen fixation in rice root tissues and >33% of the new nitrogen accumulation in younger peatlands [9, 10]. These data suggest a considerable nitrogen input via methanotrophic BNF.

Aerobic methanotrophs (namely, methane-oxidizing bacteria (MOB)) are the most common drivers of methanotrophic BNF. MOB has been found to inhabit diverse ecological niches such as the rice root, the rhizosphere, and bulk soil [11, 12]. The abundance of MOB in paddy soil was shown to follow the order: root > rhizosphere > bulk soil [13]. Such a distribution characteristic is likely due to a limited oxygen secretion and diffusion from rice roots because dissolved oxygen (DO) in the rhizosphere would gradually decrease to zero beyond a 5-mm distance from the root surface [14]. Oxygen deficiency is presumed to be as a key constraint on methanotrophic BNF in the bulk soil, as aerobic methane oxidation (AMO) depends on oxygen. Unfortunately, most previous researches on methanotrophic BNF by MOB were focused on soil-free settings with a plenty of oxygen (>2%, v/v) in the headspace [15–18], whereas methanotrophic BNF by soil MOB suffering from strong oxygen constraint has as yet been unobserved. The widespread distribution of MOB in bulk soils necessitates a further exploration on whether MOB are able to perform methanotrophic BNF under hypoxia.

Recent studies have suggested that MOB were able to perform methane oxidation under hypoxia by shifting from oxygen-dependent respiration to Fe(III)-dependent respiration (i.e. AMO coupled to Fe(III) reduction, Fe-AMO) [19, 20]. A few of the MOB, including *Methylosinus*, *Methylomonas*, and *Methylococcus*, have been reported to catalyze Fe-AMO [21–24]. The Fe-AMO process is ecologically significant as it has been suggested to predominate the methane oxidation process in iron-rich lake sediments, mitigating 40.3% of the methane emission [20]. Yet, Fe-AMO is still not correlated with methanotrophic BNF, because it was characterized under ammonium-sufficient conditions where BNF seemed unlikely to have occurred [20, 21]. Since bioavailable nitrogen is quite low in natural niches, the Fe-AMO activity evaluation under a nitrogen-deficient condition is more environmentally relevant. For the Fe-AMO process, ferrihydrite is generally used as it is the most reactive among natural iron minerals [25]. Ferrihydrite is relatively rich in the rhizosphere due to a rapid oxidation of Fe(II) [26]. The oxidation of Fe(II) often forms a layer of iron plaque (IP) that mainly (80%) consists of ferrihydrite on the rice root surface [27], providing a natural condition for rhizosphere Fe-AMO. Such a widespread coexistence of abundant ferrihydrite, methane, and MOB in the rhizosphere inspired our speculation that methanotrophic BNF may extend from the root tissues to the rhizosphere/bulk soil via ferrihydrite-mediated Fe-AMO. Such a ferrihydrite-dependent methanotrophic BNF may dissolve the IPs on the root surface, accelerating soil iron mineral transformation/transport and root nutrient uptake (e.g. nitrogen and phosphorus). The resulting Fe(II) is likely to serve as a reductant for heavy metal detoxification (such as Cr(VI)) and for the degradation of organic contaminants via the Fenton reaction.

Therefore, this work aimed to explore the possibility, performance, and mechanism of ferrihydrite-mediated methanotrophic BNF under hypoxia in the paddy soil microcosm. Methane consumption, ammonia, and Fe(II) production were monitored to verify the dependence of methanotrophic BNF on Fe-AMO. Carbon (^13^C) and nitrogen (^15^N) isotope tracing were employed to evaluate the performance of such a novel BNF process. Nitrogen-fixing and/or iron-reducing MOB were identified via DNA stable-isotope probing (DNA-SIP) and sequencing. The metabolic features associated with BNF and ferrihydrite reduction and their coupling mechanism were analyzed by metagenomics. We were able to show that Fe-AMO enhanced methanotrophic BNF rate by 13-fold, contributing significantly (81%) to total BNF. Our findings suggest MOB play an appreciable role in linking methane, nitrogen, and iron cycling in hypoxic soils.

## Materials and methods

### Soil sampling and physicochemical properties

A surface layer (top 20 cm) of bulk paddy soils was collected in October 2021 from a research farm (32°30′ N, 119°25′ E) of Yangzhou University, Jiangsu province, China, after the rice harvest [28]. The meteorological data for the farm were the same as previously described [28]. To get a sample representative of the typical paddy soil, five bulk soil subsamples in the paddy field were collected along an S-line track and were mixed to obtain a composite sample. The composite sample was transferred to the laboratory within 24 h and was divided into two parts. One part was air-dried and sieved through a 1-mm mesh, and soil physicochemical properties were determined according to standard methods [29]. Briefly, total carbon and total nitrogen were determined with an element analyzer (Vario EL Cube, Elementar, Germany); dissolved organic carbon (DOC) was determined on a total organic carbon analyzer (TOC-L CPH, Shimadzu, Japan); ammonia nitrogen was extracted with 3 M KCl for 1 h and was analyzed by the indophenol blue colorimetric method at 625 nm; total phosphorus was extracted by fusing the soil with sodium hydroxide and was quantified with the molybdenum blue method at 700 nm; total iron was extracted via digesting the soil with nitric acid-perchloric acid-hydrofluoric acid and was measured by an inductively coupled plasma optical emission spectroscopy (Avio 220 Max, PerkinElmer, USA); Fe(III) and Fe(II) were extracted with 0.5 M HCl and the supernatant/hydroxylamine hydrochloride reduced supernatant was determined by the o-phenanthroline colorimetric method at 510 nm; Fe(III) was calculated as the difference between total iron and Fe(II). Another part was preincubated at a 1:3 soil-to-ultrapure-water ratio for 1 week at room temperature to decompose organic carbon and nitrogen. The physicochemical properties of original soil and preincubated soil are summarized in Table 1.

**Table 1:** Summary of the physicochemical properties of the collected paddy soil.

Soil sample	Parameter	Value (unit)
Original soil	pH	6.61
	Total carbon	10.87 (g kg^−1^)
	Total nitrogen	1.04 (g kg^−1^)
	Nitrate nitrogen	0.71 (mg kg^−1^)
	Ammonia nitrogen	1.87 (mg kg^−1^)
	Total phosphorus	2.14 (g kg^−1^)
	Total iron	12.81 (g kg^−1^)
Preincubated soil	DOC	23.93 (mg kg^−1^)
	Nitrate nitrogen	0.60 (mg kg^−1^)
	Ammonia nitrogen	6.82 (mg kg^−1^)
	Ferric iron	1.52 (g kg^−1^)

### Methane-oxidizing bacteria enrichment and microcosm for ferrihydrite-mediated methanotrophic biological nitrogen fixation

The MOB enrichment was carried out for 160 days in a serum bottle reactor (total volume, 2.45 l; headspace, 1.22 l) (Fig. S1). Approximately, 330 g (dry weight) preincubated soil and 1 l nitrogen-free mineral salt solution (MSS, Table S1) were mixed in the reactors. Ferrihydrite was prepared using a previously described method and was amended to the reactors at an initial concentration of 10 mmol iron l^−1^ [30]. The reactors were subsequently purged with N_2_ (99.99%), aerated with CH_4_/N_2_ (20%:80%), and incubated statically in the dark at 30°C. The headspace gas (100 μl) and soil slurry (2 ml) were sampled every 10 days for CH_4_, Fe(II), and NH_4_^+^–N measurements. Every 50 days, 330 ml supernatant in each reactor was replaced with fresh MSS (300 ml) + ferrihydrite solution (30 ml) and the headspaces were reflushed with CH_4_/N_2_ (20%, 80%).

To demonstrate ferrihydrite-mediated methanotrophic BNF, the experimental treatment group (designated as CFeN) and four controls (FeN, CN, CFeNS_0_ and N) were set up in 60-ml serum bottle reactors (Table S2). The control groups were used to investigate the roles of CH_4_, ferrihydrite, MOB, and soil organics, respectively. The CFeN group was prepared by mixing 4 ml soil slurry (~1 g dry soil) from the parent reactor with autoclaved 5 ml MSS and 1 ml ferrihydrite (300 mmol l^−1^ in MSS). After the reactor was alternately filled with helium and was evacuated to make a hypoxic headspace (O_2_, 0.27% (v/v)), 10 ml CH_4_ and 40 ml N_2_ (99.99%) were injected into the headspace of evacuated reactors. The FeN group was prepared by injecting 50 ml N_2_ into a methane-free headspace as other conditions were identical with the CFeN group. The CN group was conducted by omitting ferrihydrite and supplementing with 10 ml MSS. The N group was prepared by incubating the same amount of soil slurry with 10 ml MSS under a headspace of N_2_ (50 ml) and omitting both ferrihydrite and CH_4_. A soil-free control (CFeNS_0_) was also performed identically as the CFeN group except that no soil was added. All the microcosms were run in triplicate and were incubated for 50 days at 30°C in the dark. Another batch of microcosms was conducted to confirm BNF by substituting ^15^N_2_ (purity of 99.99%) for ^14^N_2_ in CFeN, FeN, CN, and N.

### Methane oxidation inhibition and Fe(III)-limitation experiments

To evaluate the dependency of nitrogen fixation on Fe-AMO, the CFeN group with difluoromethane (CFeN + CF_2_H_2_) and the CFeN group without ferrihydrite (namely, CN) were conducted to represent AMO inhibition and Fe(III) limitation, respectively. For details, 4 ml slurry (~1 g dry soil) from the parent reactor on Day 160 was mixed with 5 ml N-free MSS and 1 ml ferrihydrite (300 mmol l^−1^) in a 60-ml reactor. The reactor was then alternately evacuated, filled with helium gas, and eventually filled with 10 ml CH_4_ and 40 ml N_2_. CH_2_F_2_, an inhibitor of methane monooxygenase [31], was added to the headspace of reactors at a final concentration of 0.5% (v/v) to achieve an inhibitory effect. The 1 ml N-free MSS was substituted for ferrihydrite to make Fe(III) limitation. There were 18 reactors for each of the three groups (CFeN, CFeN + CH_2_F_2_, and CN). Every 10 days, three reactors from each group were randomly selected to ensure the representativeness and offered to withdraw the soil slurries for Fe(II) and NH_4_^+^–N analyses. Dual isotope labeling experiments were also conducted in another parallel batch of CFeN, AMO-inhibited, and Fe(III)-limited groups with ^13^CH_4_ (10 ml) and ^15^N_2_ (40 ml) in the headspace. The soil ^13^C and ^15^N abundances were determined at the end of the incubation. The procedures for RNA extraction, cDNA synthesis by reverse transcription, and real-time quantitative PCR (qPCR) are provided in the Supplementary Information.

### DNA stable-isotope probing and real-time quantitative PCR

To identify the microorganisms involved in Fe(III)-dependent methane oxidation and nitrogen fixation and dual-isotope labeled (^13^CFe^15^N) and unlabeled (^12^CFe^14^N) treatments were performed using ^13^CH_4_ (99.99 atom%) + ^15^N_2_ (99.99 atom%) and ^12^CH_4_ + ^14^N_2_, respectively. For DNA-SIP, the batch conditions (10 ml methane and 40 ml nitrogen) were the same as mentioned above. All the reactors were subjected to a 50-day incubation at 30°C in the dark. The soil slurries (~10 ml) were then precipitated by centrifugation (8000 rpm, 15 min) to remove the supernatant. Total soil DNA was extracted by FastDNA Spin Kit for Soil (MP Biomedicals, USA) following the manufacturer’s instructions. The extracted DNA (5 μg) from the ^13^CFe^15^N and ^12^CFe^14^N groups were dissolved in CsCl solution to reach a final buoyant density (BD) of 1.714 g ml^−1^ in 4.9-ml OptiSeal polyallomer tubes, respectively. The samples were centrifugated at 53 000 rpm for 48 h at 20°C using an Optima XPN-100 Ultracentrifuge (Beckman Coulter, USA). After centrifugation, a syringe pump (LSP01-1A, Baoding Longer Precision Pump CO., Ltd, China) was applied to fractionate the obtained DNA gradients into 24 equal volumes (200 μl per fraction). The BD of each fraction was detected using a digital refractometer (AR200, Reichert, USA). Then, DNA in each fraction was precipitated with glycogen and two volumes of PEG (i.e. 400 μl), purified with 70% ethanol, and eluted with 30 μl of TE buffer. The *pmoA*, *mcrA*, and *nifH* and the bacterial and archaeal 16S rRNA genes in each fraction were quantified via a real-time PCR system (LightCycler 96, Roche, USA) using the primer pairs A189F and mb661R, mlas-mod-F and mcrA-rev-R, polF and polR, Bac515F and Bac806R, and Arch519F and Arch915R, respectively. The qPCR primer sequences, reaction mixtures, and annealing temperatures are detailed in the Supplementary Information (Table S3).

### Amplicon sequencing and shotgun metagenome sequencing

DNA sample in fractions with the top three highest *nifH* and *pmoA* gene abundances from the treatments ^13^CFe^15^N (heavy fractions) and ^12^CFe^14^N (light fractions) were subjected to amplicon sequencing of bacterial 16S rRNA and *nifH* genes with the primers listed in Table S3. The sequencing was performed on an Illumina MiSeq platform at Beijing Novogene Bioinformatics Technology Co., Ltd, China. The raw reads were processed with QIIME2 for trimming and quality filtering. After being denoised and filtered at an abundance threshold of five reads, amplicon sequence variants (ASVs) were generated with the DADA2 algorithm. The representative sequence of each ASV was annotated against the Genome Taxonomy Database (GTDB).

Shotgun metagenome sequencing was performed on an Illumina PE150 platform at Novogene Bioinformatics Technology Co., Ltd, China. Since a single heavy fraction of DNA was insufficient for metagenome sequencing, all the heavy fractions of DNA from the ^13^CFe^15^N group were pooled as one composite DNA sample. The raw sequence reads were qualified using Trimmomatic v0.36 to remove low-quality reads and host–genome contamination. A total of 147 685 624 (22.00 Gb) qualified reads were then *de novo* assembled using Megahit. Metagenomic binning was performed using MaxBIN2 and Metabat2. CheckM was used to estimate the completeness, contamination, and strain heterogeneity of the bins. Metagenome-assembled genomes (MAGs) were filtered at a threshold of completeness >80% and contamination <10%. Taxonomy annotation of the genomes and phylogenetic tree analysis were carried out using GTDB-Tk (r207) based on the GTDB. Gene annotation was performed with KOBAS 2.0 against the KEGG database with an E-value cutoff of 10^−5^. Functional genes involved in methane oxidation, BNF, and extracellular electron transfer (EET) for Fe(III) reduction were identified according to the method of Li *et al*. [20].

### Analytic techniques

CH_4_ in the headspace of reactors were determined by gas chromatography (GC) equipped with a flame ionization detector (GC-2014, Shimadzu, Japan). The initial headspace oxygen (0.27% (v/v)) was measured by GC (7890B, Agilent, USA) with an electron capture detector. DO was determined by a microelectrode using the Sensor Trace Pro software (Unisense A/S). Fe(II) in the slurry was extracted with excess 0.5 M HCl and was measured by the o-phenanthroline colorimetric method at 510 mm [32]. Ammonium was extracted with 3 M KCl for 1 h and was measured by the indophenol blue colorimetric method at 625 nm [33]. The ^15^N and ^13^C abundances in the freeze-dried soil were measured by a stable-isotope ratio mass spectrometer (Isoprime-100, Elementar). The BNF rates were calculated using the formula (SW × TN/100 × (^15^N_c1_ – ^15^N_c2_)/^15^N_g_ × 100/MW/*t*) as previously described [17], where SW is the dried soil weight (1.00 g/reactor), TN is the average nitrogen content (%, w/w), and MW is the molecular weight (30) of ^15^N_2_. The ^15^N_c1_ and ^15^N_c2_ represent the respective final and initial ^15^N concentrations (atom% excess) in the soils, respectively. The ^15^N_g_ is the ^15^N concentration (99.62 atom% excess) in the ^15^N_2_ gas, and *t* is the incubation time. The least significant difference test for geochemical parameters and microbial relative abundances in the DNA fractions were performed using the R software (version 4.3.1) package agricolae (v1.3-5).

## Results and discussion

### Evidence for ferrihydrite-mediated methanotrophic biological nitrogen fixation in soil microcosm

Three successive cycles of batch incubation with methane and ferrihydrite were conducted to enrich iron-reducing methanotrophs (i.e. MOB) under hypoxia (DO, ~0.1 mg l^−1^). During the enrichment, methane in the headspace was gradually consumed and Fe(II) increased from 18.68 ± 1.24 mmol l^−1^ on Day 0 to 28.95 ± 0.46 mmol l^−1^ on Day 50 (Fig. S1). The net Fe(II) addition (12.63 mmol) and methane consumption (2.77 mmol) gave a stoichiometric ratio of 4.56:1. This deviated from a theoretical ratio (Fe(III)/CH_4_ = 8:1) under hypoxia [20, 21], suggesting the possibility of other electron sinks (e.g. BNF). Moreover, the simultaneous enrichment of Fe(II)-oxidizing bacteria (see the DNA-SIP results below) could be also responsible for this deviation. Both methane consumption and Fe(II) generation were well reproduced in the next two batch cycles. Total NH_4_^+^–N accumulated to 0.56 ± 0.07 mmol l^−1^ during the enrichment period, exhibiting a similar trend like Fe(II) (Fig. S1). Moreover, the methane, Fe(II), and ammonium concentrations showed strong linear correlations between each other (*R*^2^ = 0.85–0.99, *P* < .01) (Fig. S2). These correlations suggested that methane consumption, ammonium production, and ferrihydrite reduction occurred in a synergetic way.

To further investigate the occurrence of ferrihydrite-mediated methanotrophic BNF, the experimental group (CFeN) was compared with four controls (FeN, CN, CFeNS_0_, N) to assess the roles of methane, ferrihydrite, MOB, and the background iron/organics, respectively. The methane consumption for CFeN was significantly higher than those for CN and CFeNS_0_ (Table 2), demonstrating ferrihydrite-enhanced methane utilization by MOB. Meanwhile, more Fe(II) and ammonium were produced in CFeN than in FeN, CN, and N groups (*P* < .05) (Table 2). The generated Fe(II) strongly correlated with the accumulated ammonium in the five groups (*R*^2^ = 0.94, *P* < .001) (Fig. S2), indicating that ammonification or BNF was coupled to ferrihydrite reduction [34].

**Table 2:** Net methane, Fe(II) and ammonium changes as well as the soil ^15^N abundances and BNF rates for different treatment groups after a 50-day incubation.

Group	Methane change (μmol)	Ferrous ion increment (μmol)	Ammonium production (μmol)	^15^N abundance in soils (atom%)	^15^N fixed (atom% excess)	BNF rate (μmol-N_2_ g_dw_^−1^ d^−1^)
CFeN	−59.10 ± 1.53 a	75.67 ± 1.10 a	1.14 ± 0.06 a	0.542 ± 0.045 a	0.173 ± 0.045 a	0.283 ± 0.052 a
CN	−22.55 ± 1.21 b	27.45 ± 2.80 c	0.89 ± 0.01 b	0.376 ± 0.001 b	0.007 ± 0.001 b	0.010 ± 0.002 b
FeN	0.15 ± 0.05 e	56.54 ± 1.35 b	0.92 ± 0.04 b	0.403 ± 0.000 b	0.034 ± 0.000 b	0.047 ± 0.004 b
N	0.09 ± 0.01 e	16.59 ± 0.65 d	−0.20 ± 0.03 e	0.382 ± 0.015 b	0.013 ± 0.015 b	0.016 ± 0.018 b
CFeNS_0_	−8.59 ± 1.09 c	0.22 ± 0.05 e	0.09 ± 0.04 d	—	—	—
CFeN+CF_2_H_2_	−3.07 ± 0.72 d	27.74 ± .19 c	0.71 ± 0.04 c	0.410 ± 0.004 b	0.041 ± 0.004 b	0.045 ± 0.017 b
Estimated Fe(III)-mediated methanotrophic N_2_ fixation						0.238^a^

a calculated from the BNF rate (per g dry weight of soil) difference between the treatments CFeN and CFeN+CF_2_H_2_; net changes for methane, Fe(II) and ammonium were obtained by subtracting the data for day 0.

— denotes data not detected.

^15^N_2_ isotope tracing and the soil ^15^N abundance (^15^N/(^15^N + ^14^N)) measurement were conducted to verify BNF. CFe^15^N exhibited a much higher soil ^15^N abundance (0.565 ± 0.018 atom%) than those for Fe^15^N, C^15^N and ^15^N (Table 2). By contrast, the CFe^14^N group showed no increase in soil ^15^N abundance (0.369 ± 0.000 atom%), ruling out the possibility of nitrogen isotope fractionation [35]. Hence, the enhanced ^15^N_2_ assimilation by the presence of both methane and ferrihydrite confirmed the occurrence of Fe(III)-mediated methanotrophic BNF. The slight increase (0.034 atom%) in the ^15^N abundance in Fe^15^N might be driven by the degradation of soil organics [36, 37]. Compared to CFeN, the high Fe(II) and ammonium production with a low BNF activity in Fe^15^N suggested that Fe(III) reduction was preferentially coupled to ammonification of nitrogen-containing organics rather than BNF in this group. These results indicated BNF in CFeN was primarily attributed to methane and to a lesser extent to soil organics. It was therefore inferred that methane-fueled ferrihydrite reduction played a predominant role in BNF.

### Dependence of biological nitrogen fixation on methane oxidation and Fe(III) reduction

**Figure 1:**
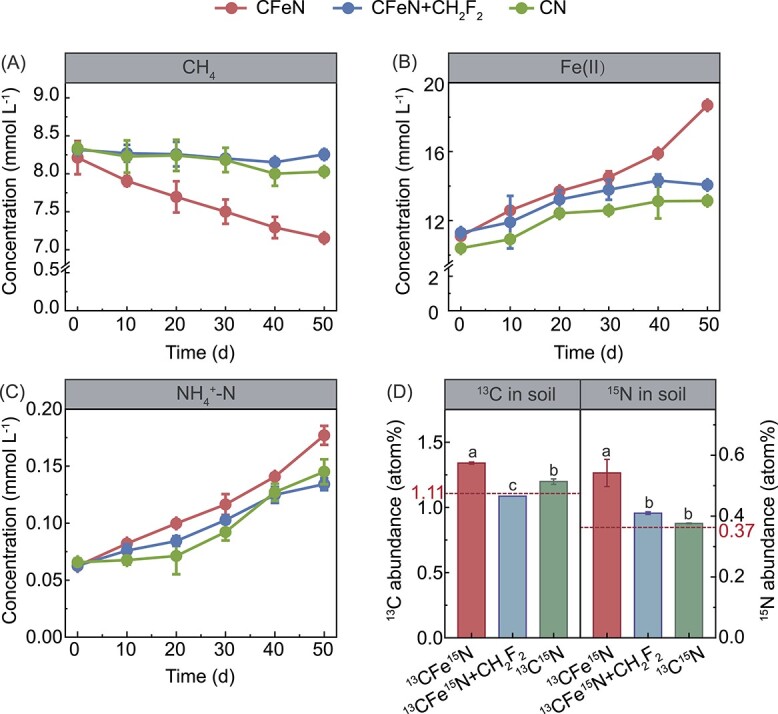
Kinetics of methane consumption (A), production of Fe(II) (B), and ammonium (C) in the CFeN, CFeN + CF_2_H_2_, and CN groups; the incorporated ^13^C and ^15^N abundances in paddy soils incubated under the dual-isotope-labeled condition for 50 days (D); data are mean ± SD of three independent replicates (*n* = 3).

To clearly demonstrate the dependence of BNF on methane oxidation and Fe(III) reduction, AMO-inhibited and Fe(III)-limited experiments were performed by adding CF_2_H_2_ and omitting ferrihydrite, respectively. As shown in Fig. 1A, the methane concentration in the CFeN group decreased significantly over time. By contrast, methane was hardly consumed in the CFeN+CF_2_H_2_ group, confirming that AMO was responsible for methane consumption. Meanwhile, more Fe(II) and ammonium (18.69 and 0.18 mmol l^−1^, respectively) accumulated in CFeN compared to those in CFeN+CF_2_H_2_ (14.07 and 0.13 mmol l^−1^, respectively) (Fig. 1B and C), verifying that AMO had led to Fe(III) reduction and ammonium production. The small Fe(II) increment (2.77 mmol l^−1^) in CFeN + CF_2_H_2_ indicated that nonmethanotrophic processes (e.g. degradation of soil organics) contributed only a minor part to Fe(III) reduction.

Isotope tracing with ^13^CH_4_ and ^15^N_2_ showed that the soil ^13^C abundance (^13^C/(^13^C + ^12^C), 1.340 ± 0.008 atom%) for CFeN was higher than the natural background value (1.108 atom%) (Fig. 1D), suggesting the ^13^CH_4_ assimilation by soil microorganisms. Contrarily, no ^13^CH_4_ assimilation was detected for CFeN + CF_2_H_2_. Likewise, the soil ^15^N abundance for CFeN (0.542 ± 0.045 atom%) was significantly higher than that (0.410 ± 0.004 atom%) of CFeN + CF_2_H_2_ (*P* < .05) (Fig. 1D). Thus, the amendment of methane and ferrihydrite enhanced the BNF rate by 13-fold from 0.02 to 0.28 μmol N_2_ g_dw_^−1^ d^−1^ (soil dry weight), which was close to that of moss-associated MOB (Table 3). By contrast, the BNF rate for the CFe^15^N + CF_2_H_2_ group was only 0.05 μmol N_2_ g_dw_^−1^ d^−1^. This indicated that ferrihydrite-mediated methanotrophic BNF (0.24 μmol N_2_ g_dw_^−1^ d^−1^) accounted for 81.1% of the total BNF, whereas the remainder (18.9%) was attributable to nonmethanotrophic BNF. The Fe-AMO based methanotrophic BNF rate was 48.6% of that (0.49 μmol N_2_ g_dw_^−1^ d^−1^) for the rice root (Table 3) [10], likely due to the greater oxygen constraint on AMO in the soil. Based on these results, it was concluded that AMO rather than soil organics was mainly responsible for Fe(III) reduction and BNF.

**Table 3:** Comparison of Fe(III)-mediated methanotrophic BNF with previous studies.

Diazotrophs	Habitat	Electron acceptor (concentration)	Methanotrophic BNF rate (μmol-N_2_ g_dw_^−1^ d^−1^)	Reference
*Methylocystis/Methylosinus*	Rice root	Oxygen (5%, v/v)	0.49	[10]
*Methylomonas*	Rice root	Oxygen (10%, v/v)	0.72^a^	[18]
*Methylosinus*	*S. triqueter* root	Oxygen (5%, v/v)	134.40	[17]
Unknown methanotrophs	*Sphagnum* moss	Oxygen (—)	0.34^b^	[9]
ANME-2	Sediment	Sulfate (—)	1.27 × 10^–3 c^	[63]
*Methylosinus* sp. 3S-1	Culture medium	Oxygen (10%, v/v)	2256^d^	[31]
*Methylocystis*/*Methylomicrobium*, *Methylophilaceae*	Rice paddy	Ferrihydrite (10 mM)	0.24	This study

a Calculated from the fixed nitrogen content of 43.3 μmol N g_dw_^−1^ over 30 days.

b Calculated by subtracting the nonmethanotrophic BNF rate (35 nmol N_2_ g_dw_^−1^ h^−1^) from the BNF rate (49 nmol N_2_ g_dw_^−1^ h^−1^) in the presence of light and methane.

c Based on the net BNF (327.8 nmol N_2_ g_dw_^−1^) in Hydrate Ridge Seep (Mat-774) at a depth of 3–6 cm in the presence of methane over 258 days.

d Normalized to the bacterial dry weight.

— denotes data not available.

**Figure 2:**
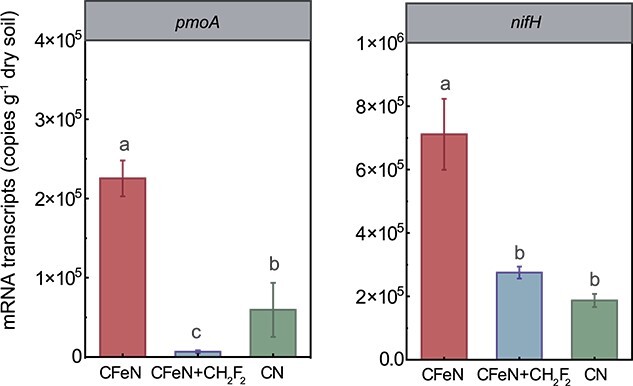
Comparison of the *pmoA* and *nifH* mRNA transcripts in the CFeN, CFeN + CF_2_H_2_, and CN groups on Day 20 of incubation; data are mean ± SD of three independent replicates (*n* = 3).

After a 50-day incubation, 0.31 mmol l^−1^ of methane was consumed with simultaneous increases of 2.75 mmol l^−1^ of Fe(II) and 0.08 mmol l^−1^ of ammonium for CN, which was lower than those for CFeN (Fig. 1). This indicated that the endogenous Fe(III) in CN (4.10 mmol l^−1^) was not enough or available (e.g. iron oxide surface enveloped by soil components) to support an appreciable methane utilization, namely a Fe(III) limitation effect was achieved. At the RNA level, the CFeN group showed higher *pmoA* and *nifH* transcript abundances compared with CN (Fig. 2), agreeing with previous findings that Fe(III) fertilizer increased the potential activity of methanotrophs in rice paddies and that methane-fueled Fe(III) reduction promoted the expression of nitrogen-fixing enzymes in *Methanosarcina barkeri* [38, 39]. The enhanced gene transcription could be due to the reasons that (i) ferrihydrite provided the electron acceptor for Fe-AMO and (ii) ferrihydrite likely induced the oxygen generation by methanobactins to alleviate the oxygen constraint [40]. The isotope tracing for the ^13^C^15^N group showed the soil ^13^C abundance increased from a background value (1.108 atom%) to 1.198 atom%, whereas the soil ^15^N abundance increment (0.007 atom%) was negligible (Fig. 1D). The corresponding BNF rate for ^13^C^15^N (0.01 μmol N_2_ g_dw_^−1^ d^−1^) was only 3.5% of that for ^13^CFe^15^N (~0.28 μmol N_2_ g_dw_^−1^ d^−1^) (Table 2). In other words, ferrihydrite-mediated BNF accounted for 96.5% of the total BNF in ^13^CFe^15^N, confirming the dependence of BNF on ferrihydrite reduction. Taken together, these findings demonstrated for the first time that BNF was driven by Fe-AMO.

### Identification of functional microorganisms through DNA stable-isotope probing

**Figure 3:**
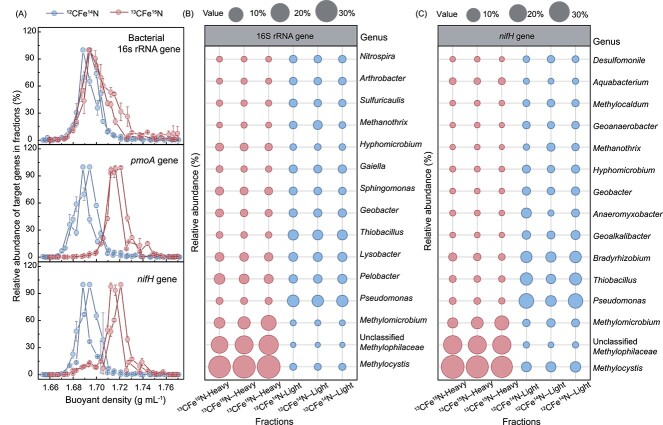
Normalized relative abundances of target genes in the gradient ultracentrifugation fractions of heavy ^13^CFe^15^N-DNA and light ^12^CFe^14^N-DNA (A) and microbial community compositions in the ^13^CFe^15^N and ^12^CFe^14^N groups based on 16S rRNA (B) and *nifH* (C) sequencing.

DNA-SIP was used to reveal the microorganisms which had assimilated the isotope ^13^C and/or ^15^N. The DNA samples from microcosms fed with ^13^CH_4_ + ^15^N_2_ and ^12^CH_4_ + ^14^N_2_ (namely ^13^CFe^15^N-DNA and ^12^CFe^14^N-DNA, respectively) were fractionated by CsCl gradient ultracentrifugation. The bacterial and archaeal 16S rRNA, *pmoA*, *nifH*, and *mcrA* genes in the fractions were quantified via qPCR. Compared with the ^12^CFe^14^N-DNA fractions, the ^13^CFe^15^N-DNA fractions showed a higher relative abundance of bacterial 16S rRNA genes at the heavy BD of 1.72 g ml^−1^ (Fig. 3A). This implied that certain bacteria had assimilated the isotope (^13^C and/or ^15^N). For the archaeal 16S rRNA and *mcrA* genes, ^13^CFe^15^N-DNA and ^12^CFe^14^N-DNA were distributed in a similar pattern across the entire BD with single peaks, which overlapped well around the light BD of 1.69 g ml^−1^ (Fig. S3). Thus, archaea were not active in isotope uptake. However, *pmoA* and *nifH* in ^13^CFe^15^N-DNA fractions exhibited a major peak at the heavy BD of 1.72 g ml^−1^, whereas these genes in ^12^CFe^14^N-DNA fractions peaked at the light BD of 1.69 g ml^−1^. Such a peak shift toward the heavy BD indicated that methanotrophs and diazotrophs were involved in isotope (^13^C and/or ^15^N) incorporation [41]. Similarly, *Geobacter* had a higher abundance at the BD of 1.70 g ml^−1^ in the heavy fractions (^13^CFe^15^N-DNA) than in the light fractions (^12^CFe^14^N-DNA) (Fig. S3C), demonstrating that *Geobacter* had assimilated the isotope ^13^C and/or ^15^N, namely, *Geobacter* participated in iron reduction and/or nitrogen fixation using soil organic matter or ^13^CH_4_-derived products as the substrate. This may account for the observed nonmethanotrophic BNF activity in the group CFeN+CF_2_H_2_.

The heavy ^13^CFe^15^N-DNA and light ^12^CFe^14^N-DNA fractions containing the top three highest *nifH* abundances were subjected to 16S rRNA and *nifH* sequencing. *Methylocystis* (30.18% ± 2.09%) dominated in the heavy ^13^CFe^15^N-DNA fractions and was significantly more abundant than in light fractions (0.32% ± 0.04%) (Fig. 3B). *Methylocystis* is a facultative Type II methanotroph and has a demonstrated capacity of methanotrophic BNF [42–44]. *Methylocystis* was therefore considered to be a key diazotrophic methanotroph in our microcosms. Due to the enrichment in heavy fractions, unclassified *Methylophilaceae* (17.16% ± 3.55%) and *Methylomicrobium* (6.08% ± 3.57%) were also identified as putative methylotroph/methanotroph and/or diazotrophs. The family *Methylophilaceae* is associated with methylotrophs unable to grow on methane [45]. The enrichment of *Methylophilaceae* in the heavy fractions suggested that it probably incorporated ^13^CH_4_ derived intermediates (e.g. methanol) and/or ^15^N_2_. Some Fe(II)-oxidizing bacteria were also present in the light/heavy fractions, including *Pseudomonas* (4.10%/0.34%), *Thiobacillus* (2.32%/0.20%), *Bradyrhizobium* (0.10%/0.15%), and *Hyphomicrobium* [46–49]. Moreover, diazotrophic *Hyphomicrobium* was enriched in heavy fractions (0.69% ± 0.16%) than in light ones (0.20% ± 0.01%). These suggested the produced Fe(II) from Fe-AMO could be reoxidized by these Fe(II)-oxidizing bacteria for ^13^C/^15^N incorporation, accounting for a lower stoichiometric ratio for Fe-AMO.

Similarly, *nifH* sequencing showed that the relative abundances of *Methylocystis* (35.01% ± 3.41%), unclassified *Methylophilaceae* (21.11% ± 0.57%), and *Methylomicrobium* (6.13% ± 3.49%) in the heavy fractions (^13^CFe^15^N-DNA) were higher than those (2.92% ± 0.42%, 0.12% ± 0.06%, 0.89% ± 0.30%, respectively) in the light fractions (^12^CFe^14^N-DNA) (Fig. 3C), confirming that these taxa were involved in methane oxidation and/or nitrogen fixation. Methanotrophs, methylotrophs, and canonical Fe(III)-reducing bacteria (*Geobacter* and *Anaeromyxobacter*) were potential ferrihydrite reducers in our microcosms [21, 50, 51]. Although *Geobacter* (0.95%) and *Anaeromyxobacter* (1.24%) were not enriched in the heavy fractions compared to light ones (Fig. 3C), their participation in Fe(III) reduction could not be ruled out. Therefore, *Methylocystis*, *Methylophilaceae*, and *Methylomicrobium* are predicted to either reduce Fe(III) independently for survival or cooperate with Fe(III)-reducing bacteria (e.g. *Geobacter*) [51, 52]. In addition, the Fe(II)-oxidizing bacteria *Pseudomonas* (8.35%), *Thiobacillus* (4.72%), and *Bradyrhizobium* (4.14%) were abundant in the nitrogen-fixing community, suggesting their involvement in Fe(III)/Fe(II) cycling.

### Metabolic potential of putative diazotrophic methanotrophs

**Figure 4:**
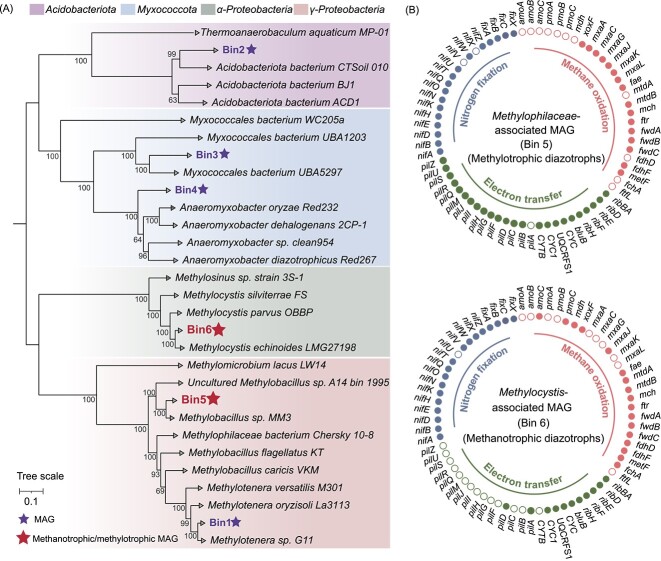
A genome-wide phylogenetic tree showing taxonomic positions of recovered MAGs (A) and the genes for methane oxidation, nitrogen fixation, and electron transfer in the MAGs associated with *Methylocystis* and *Methylophilaceae* (B).

Metagenomic sequencing and binning were conducted to predict the metabolic potential of Fe(III) reduction and BNF by methanotrophs. A total of 23 bins were gained and classified as “Bacteria.” Fortunately, six high-quality MAGs (completeness >80% and contamination <10%) were recovered (Table S4). Among them, two MAGs associated with methane/methanol oxidation were identified as *Methylophilaceae* (Bin 5) and *Methylocystis* (Bin 6). The remaining four MAGs were classified as *Methylotenera*, *Thermoanaerobaculi*, *Myxococcales*, and *Anaeromyxobacter*. A genome-wide phylogenetic tree was constructed to reveal the taxonomic positions of MAGs (Fig. 4A). The *Methylophilaceae* MAG (Bin 5, 88.1% completeness and 1.2% contamination) was phylogenetically clustered to *Methylobacillus* sp. MM3 with an orthologous average nucleotide identity (ANI) of 81.3% between them (Fig. 4A). For the *Methylocystis* MAG (Bin 6, 93.1% completeness and 1.7% contamination), the phylogenetically closest relative was *Methylocystis echinoides* with an ANI of 84.0%. This was lower than the threshold ANI (95%) demarcating different species [53], suggesting that it is possibly a novel *Methylocystis* sp.

**Figure 5:**
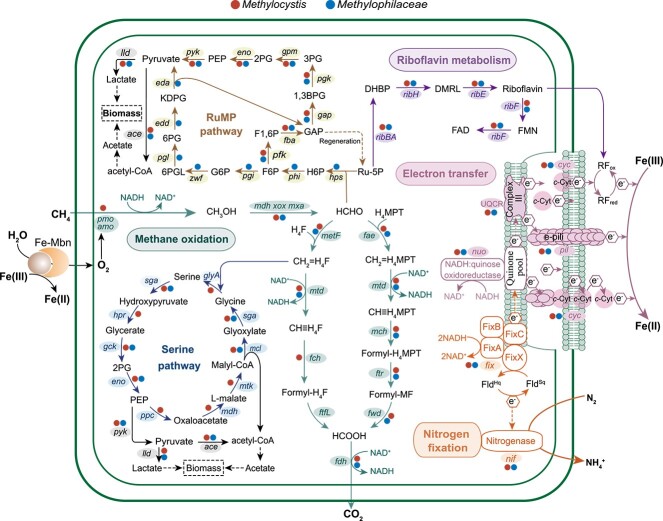
Proposed metabolic pathway for Fe(III)-mediated methanotrophic BNF by the methanotroph/methylotroph; Ru-5P: ribulose 5-phosphate, H6P: hexose 6-phosphate, F6P: fructose 6-phosphate, F1,6P: fructose 1,6-bisphosphate, GAP: glyceraldehyde 3-phosphate, 1,3-BPG: 1,3-bisphosphoglycerate, 3PG: 3-phosphoglycerate, 2PG: 2-phosphoglycerate, PEP: phosphoenolpyruvate, G6P: glucose-6-phosphate, 6PGL: 6-phosphogluconolactone, 6PG: 6-phosphogluconate, KDPG: 2-keto-3-deoxy-6-phosphogluconate, DHBP: 3,4-dihydroxy-2-butanone 4-phosphate, DMRL: 6,7-dimethyl-8-ribityllumazine, FMN: flavin mononucleotide, FAD: flavin adenine dinucleotide, Fld^Hq^/Fld^Sq^: flavodoxin hydroquinone/semiquinone, RF_ox_/RF_red_: oxidized/reduced riboflavin, H_4_F: tetrahydrofolate, H_4_MPT: methenyltetrahydromethanopterin, Fe-Mbn: Fe-bound methanobactin.

Particulate methane monooxygenase and soluble methane monooxygenase, encoded by the *pmoCAB* operon and *mmoXYBZDC* operon, respectively, are two central enzymes for converting methane to methanol [54]. *pmoC* and a complete set of gene encoding functions associated with conversion of methanol to CO_2_ were detected in the *Methylocystis* MAG (Figs 4B and 5), supporting its identity as a methanotroph. Both *pmoA* and *pmoB* were missing, presumably due to the MAG incompleteness. In addition, the partially present RuMP pathway, the serine cycle assimilating formaldehyde into biomass, and the biosynthetic pathways of acetate, lactate, and pyruvate were detected in the MAG. Thus, methane-derived intermediates (methanol, etc.) from *Methylocystis* could support the coexistence of accompanying members. For the *Methylophilaceae* MAG, all the genes encoding enzymes for methanol oxidation, except *mdh* and *mtdA* and a complete RuMP pathway, were present in this MAG (Fig. 5). Therefore, *Methylocystis* and *Methylophilaceae* could cooperate syntrophically during Fe-AMO.

The *Methylocystis* and *Methylophilaceae* MAGs possessed all the necessary genes encoding the riboflavin biosynthesis pathway (Fig. 5 and Fig. S4), suggesting that they had the potential of ferrihydrite reduction via riboflavin-mediated EET. To support this statement, the filtrate of the CFeN treatment was analyzed by fluorescence spectrometry and differential pulse voltammetry (DPV). The spectra showed two excitation peaks at 370 and 445 nm and an emission peak at 525 nm (Fig. S5), demonstrating the presence of riboflavin in the filtrate [55]. DPV analyses showed that a redox-active species with a midpoint potential of −173 mV versus the standard hydrogen electrode was present in the filtrate of the CFeN soil slurry (Fig. S6), which could be assigned to riboflavin [55]. *c*-type cytochromes (*c*-Cyts) and conductive pili are well known to mediate electron transfer to Fe(III) [56, 57]. For example, *Methylophilus* (a genus of *Methylophilaceae*) was previously demonstrated to transfer electrons extracellularly to ferrihydrite, which was attributed to the mediation of *c*-Cyts and riboflavin [50]. Both *Methylophilaceae* and *Methylocystis* MAGs contained the genes encoding *c*-Cyts and pili. Thus, *Methylophilaceae* and *Methylocystis* might utilize *c*-Cyts, riboflavin, and conductive pili for ferrihydrite reduction. Alternatively, the methanobactins of methanotrophs (e.g. *Methylocystis*) could also produce Fe(II) [40]. A further study is needed to distinguish the effect of methanobactins on ferrihydrite reduction.

A *nif* gene cluster of the *Methylocystis* MAG was identified in a 11-kb nitrogen fixation region (Fig. S7). The *nif* cluster contained 23 genes, including conserved genes *nifH*, *nifD*, nifK, *nifB*, *nifE*, and *nifN*, that have been shown to be essential for BNF. These *nif* genes were organized in an order identical with those of diazotrophic methanotrophs *M. echinoides* and *Methylosinus* sp. 3S-1, but these were oriented in the opposite direction. Similarly, the genes encoding nitrogenase (*nifK*), activation proteins (*nifZ*, *nifW*, and *nifU*), nitrogenase cofactor synthesis (*nifB*, *nifE*, and n*ifQ*), and genes with unknown functions (*nifT*) were present in the *Methylophilaceae* MAG [58]. This indicated that *Methylocystis* and *Methylophilaceae* had the potential of BNF. The *nif* gene cluster was flanked by a *fix* gene cluster encoding the electron bifurcating protein complex FixABCX (Fig. S7). As a result, *Methylocystis* sp. (Bin 6) is predicted to be using FixABCX to donate electrons to nitrogenase, as FixABCX was previously suggested to serve as an electron transport chain for BNF in diazotrophs (e.g. *Azotobacter vinelandii*) [59].

Based on the metagenomic results, a putative metabolic pathway linking methane oxidation to ferrihydrite reduction and BNF by methanotrophs could be identified (Fig. 5). Methane was oxidized to CO_2_ where the required oxygen atoms were likely derived from the residual DO or water splitting by Fe(III)-methanobactins [40]. The generated reducing equivalent NADH in this process carried electrons to NADH: quinone oxidoreductase and FixABCX. While one part of the electrons was transferred to the quinone pool by these two complexes, the other part was diverted to nitrogenases for BNF. Multi-heme cytochromes in the outer membrane accepted electrons from the quinone pool via Complex III in a final step and used them for direct or riboflavin-mediated ferrihydrite reduction. Alternatively, ferrihydrite-released Fe(III) was likely reduced by methanobactins of MOB (e.g. *Methylocystis*) via water splitting [40], providing dioxygen for the initial methane oxidation/activation (i.e. 2CH_4_ + O_2_ → 2CH_3_OH).

### Environmental implications

Microbial Fe(III)/Fe(II) cycling plays a fundamental role in biogeochemical reactions, and dissimilatory Fe(III) reduction (DIR) has been shown to be central to many environmental processes [60, 61]. The present work revealed the capability of *Methylocystis* and *Methylomicrobium* to function as new Fe(III) reducers, suggesting a diversity of MOB participating DIR in hypoxic paddy soil. The results also suggested that Fe-AMO is able to occur in nitrogen-deficient environments. Although Fe(III) may be depleted by DIR, an alternation of oxic and anoxic conditions by wetting and drying was shown to regenerate Fe(III) to maintain Fe-AMO. The observed BNF driven by Fe-AMO indicates a novel coupling pathway of C, N, and Fe cycling. Nitrogen deficiency is often a limiting factor in soil methane oxidation [62]. Fe-AMO coupled BNF is therefore able to alleviate nitrogen constraints, promoting methane oxidation. The fixed nitrogen provides an alternative explanation for unidentified nitrogen sources in soil systems [8] and suggests that iron application may be feasible to improve the soil fertility.

### Conclusions

Our results demonstrate that methane oxidation coupled to ferrihydrite reduction was able to significantly (by 13-fold) enhance the BNF rate. Ferrihydrite-mediated methanotrophic BNF contributed 81% to the total BNF in hypoxic paddy soil. *Methylocystis, Methylophilaceae*, and *Methylomicrobium* were the predominant methanotrophs/methylotrophs that were responsible for Fe-AMO-coupled BNF. These microbes likely reduced ferrihydrite independently via riboflavin and *c*-Cyts or cooperated with other iron-reducing bacteria (e.g. *Geobacter*). Future research should be devoted to exploring the *in situ* activity and distribution of this novel BNF across Fe(III)-rich rice paddies. We suggest that future research also comprehensively explore the microbial community structure characteristics of iron-mediated methanotrophic BNF in different regions and the interactions between Fe(II)-oxidizing bacteria and methanotrophs. A further understanding of the influencing factors (such as soil organic carbon, fertilization, and the alternation of wetting and drying) is imperative to understand and predict the contribution of iron-mediated methanotrophic BNF to nitrogen accumulation in paddy soils.

## Supplementary Material

Supplementary_material_ycae030

## Data Availability

The raw amplicon sequences and metagenomic data were deposited to the NCBI Sequence Read Archive under the BioProject number PRJNA1026789 and PRJNA1026874, respectively.
