# Supplementary material for: Ferrihydrite-mediated methanotrophic nitrogen fixation in paddy soil under hypoxia
Source: ISME Commun. 2024 Mar 4;4(1):ycae030. doi: 10.1093/ismeco/ycae030 (PMC10960957; doi:10.1093/ismeco/ycae030)
Supplement: Supplementary_material_ycae030 [file supplementary_material_ycae030.pdf]

Supplementary information

Ferrihydrite-Mediated Methanotrophic Nitrogen Fixation in Paddy Soil under Hypoxia

Linpeng Yu,[a,*] Rong Jia,[a,b,*,#] Shiqi Liu,[a] Shuan Li,[a] Sining Zhong,[a] Guohong Liu,[c] Raymond Jianxiong Zeng,[a] Christopher Rensing,[a] Shungui Zhou[a,#]

[a] Fujian Provincial Key Laboratory of Soil Environmental Health and Regulation, College of Resources and Environment, Fujian Agriculture and Forestry University, Fuzhou 350002, China

[b] Key Laboratory of Land Resources Evaluation and Monitoring in Southwest China, Ministry of Education, Sichuan Normal University, Chengdu, Sichuan Province 610066, China

[c] Agricultural Bio-resources Research Institute, Fujian Academy of Agricultural Sciences, Fuzhou 350003, China

[*] These authors contributed equally to this work.

[#] Corresponding author: Shungui Zhou, email: sgzhou@soil.gd.cn; Rong Jia, email: rongjiasicnu@163.com

Tel: +86 591 86397843, Fax: +86 591 86397843

Numbers of Pages: 10

Numbers of Tables: 4

Numbers of Figures: 7

RNA extraction, cDNA synthesis and qPCR experiments. Soils from the CFeN, CFeN+CF_2H_2 and CN groups were sampled on day 20 of the incubations to extract soil RNA with the E.Z.N.A. Soil RNA Mini Kit (OMEGA Bio-tek, China) according to the manufacturer's instructions. The extracted RNA were used for reverse transcription and cDNA synthesis using TaKaRa PrimeScript II 1st Strand cDNA Synthesis Kit (TaKaRa Bio Inc, Japan) following the manufacturer's protocol. cDNA and the fractionated DNA from DNA-SIP experiments were used as the templates for qPCR and the primers are provided in Table S3. The qPCR reaction mixture contained 10 μL of iTaq Universal SYBR Supermix, 0.3 μL of each primer (10 μM), 1 μL of template DNA and was filed up to 20 μL with sterilized water. For bacterial and archaeal 16S rRNA genes, the amount of each primer was 0.6 μL. The qPCR procedure was: initial denaturation at 95°C for 5 min, followed by 40 cycles of 95°C for 30 s, respective annealing temperature for 45 s and 72°C for 45 s. The annealing time was adjusted to 30 s for the archaeal 16S rRNA gene. The calibration curve for qPCR was generated using a serially diluted plasmid standard containing the targeted fragment.

The riboflavin detection by fluorescence spectrophotometry and differential pulse voltammetry (DPV). The supernatant from the 2.45-L reactor was filtrated with a membrane filter (pore diameter, 0.22 μm). The filtrate was scanned by fluorescence spectrophotometry using a standard riboflavin solution as a reference. The fluorescence spectra were recorded using the Agilent Cary Eclipse Fluorescence Spectrometer (Agilent Technologies, California, USA). The excitation spectra and emission spectra for the filtrate were determined according to a previously described method at an emission wavelength of 525 nm and at an excitation wavelength of 445 nm, respectively [1]. DPV of the filtrate was determined with an

electrochemical workstation (CHI440C, Chenhua, Co., Ltd., Shanghai, China) in a 30-mL electrochemical cell under a nitrogen gas atmosphere. A glassy carbon electrode (5 mm in diameter), platinum mesh electrode (1cm^2) and saturated calomel electrode (SCE) were used as the working electrode, counter electrode and reference electrode, respectively. The parameters for DPV were: potential increment, 4 mV; amplitude, 50 mV; pulse width, 0.2 s; sampling width, 0.02 s; pulse period, 0.5 s. The scan potential range was between -0.4 and +0.4 V vs. standard hydrogen electrode (SHE).

Table S1 The components of nitrogen-free mineral salt solution (MSS)

**Component**	**Concentration (L^{-1})**
$MgSO_4 \cdot 7H_2O$	1.00 g
$CaCl_2 \cdot 2H_2O$	0.20 g
K_2HPO_4	0.70 g
KH_2PO_4	0.54 g
Trace element solution	1 mL

The trace element solution

**Component**	**Concentration (g L^{-1})**
$ZnSO_4 \cdot 7H_2O$	0.10
$MnCl_2 \cdot 4H_2O$	0.03
H_3BO_3	0.30
$CoCl_2 \cdot 6H_2O$	0.20
$CuCl_2 \cdot 2H_2O$	0.01
$NiCl_2 \cdot 6H_2O$	0.02
$Na_2MoO_4 \cdot 2H_2O$	0.06

Table S2 Experimental setup for the five different treatment groups.

Treatment group	Methane	Ferrihydrite	Paddy Soil
CFeN	+	+	+
CN	+	–	+
FeN	–	+	+
N	–	–	+
$CFeNS_0$	+	+	–

+: supplemented; –: not supplemented.

Table S3 Primers used in this study

Gene	Primer	Sequence (5' to 3')	Annealing temperature (°C)
Bacterial 16S rRNA	Bac515F	GTGCCAGCMGCCGCGG	57
	Bac806R	GGACTACNVGGGTWTCTAAT	
Archaeal 16S rRNA	Arch519F	CAGCCGCCGCGGTAA	61
	Arch915R	GTGCTCCCCCGCCAATTCCT	
pmoA	A189F	GGNGACTGGGACTTCTGG	60
	mb661R	CCGGMGCAACGTCYTTACC	
nifH	polF	TGCGAYCCSAARGCBGACTC	60
	polR	ATSGCCATCATYTCRCCGGA	
mcrA	mlas-mod-F	GGYGGTGTMGGDTTCACMCARTA	60
	mcrA-rev-R	CGTTCATBGCGTAGTTVGGRTAGT	
Geobacter 16S rRNA	Geo494F	AGGAAGCACCGGCTAACTCC	55
	Geo825R	TACCCGCRACACCTAGT	

Table S4 Properties of six recovered MAGs with completeness >80% and contamination <10%

ID	Contig number	Size (Mbp)	Completeness (%)	Contamination (%)	Heterogeneity (%)	Taxonomic identity
bin1	290	2.0	89.2	1.4	16.7	*Methylotenera*
bin2	411	3.2	84.7	5.2	25.0	*Thermoanaerobaculia*
bin3	407	7.9	89.0	7.0	6.7	*Myxococcales*
bin4	307	4.2	89.9	2.9	20.0	*Anaeromyxobacter*
bin5	314	2.4	88.1	1.2	42.9	*Methylophilaceae*
bin6	59	3.3	93.1	1.7	50.0	*Methylocystis*

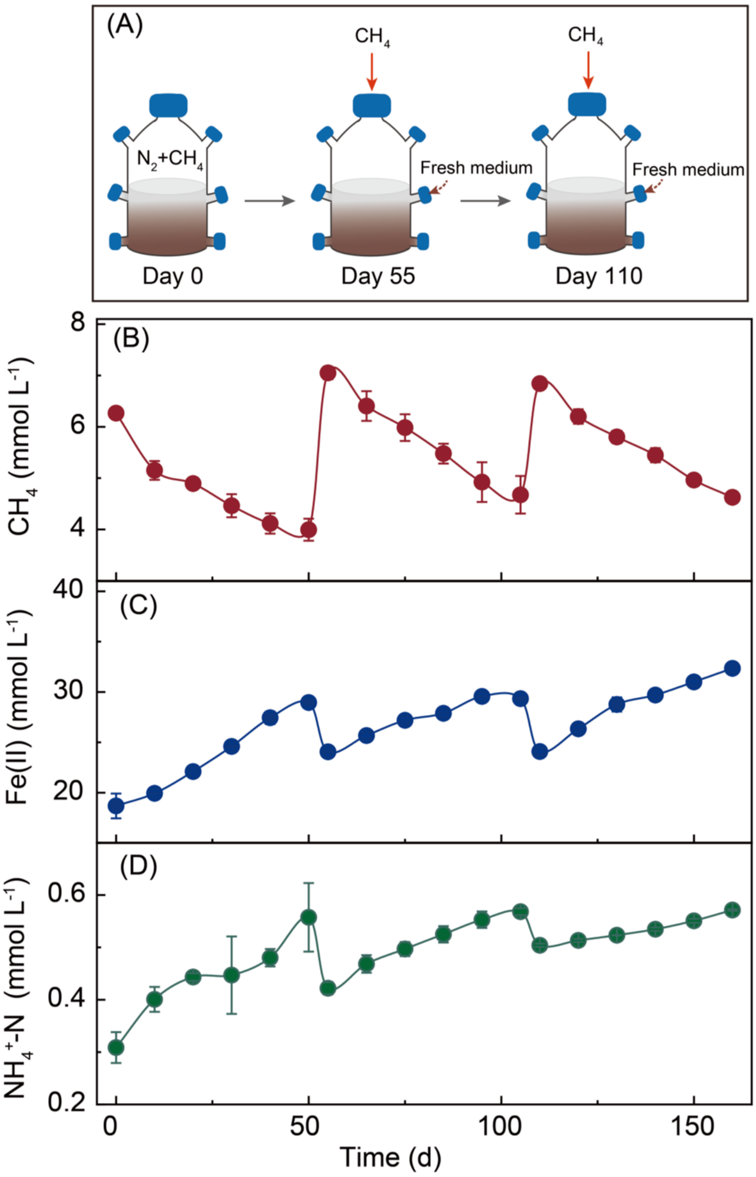

Figure S1 Experimental setup for the MOB enrichment in paddy soils incubated with methane

and ferrihydrite (A). Methane consumption (B), Fe(II) (C) and ammonium production (D) during the enrichment period. Data are mean ± SD of three independent biological replicates (n =3).

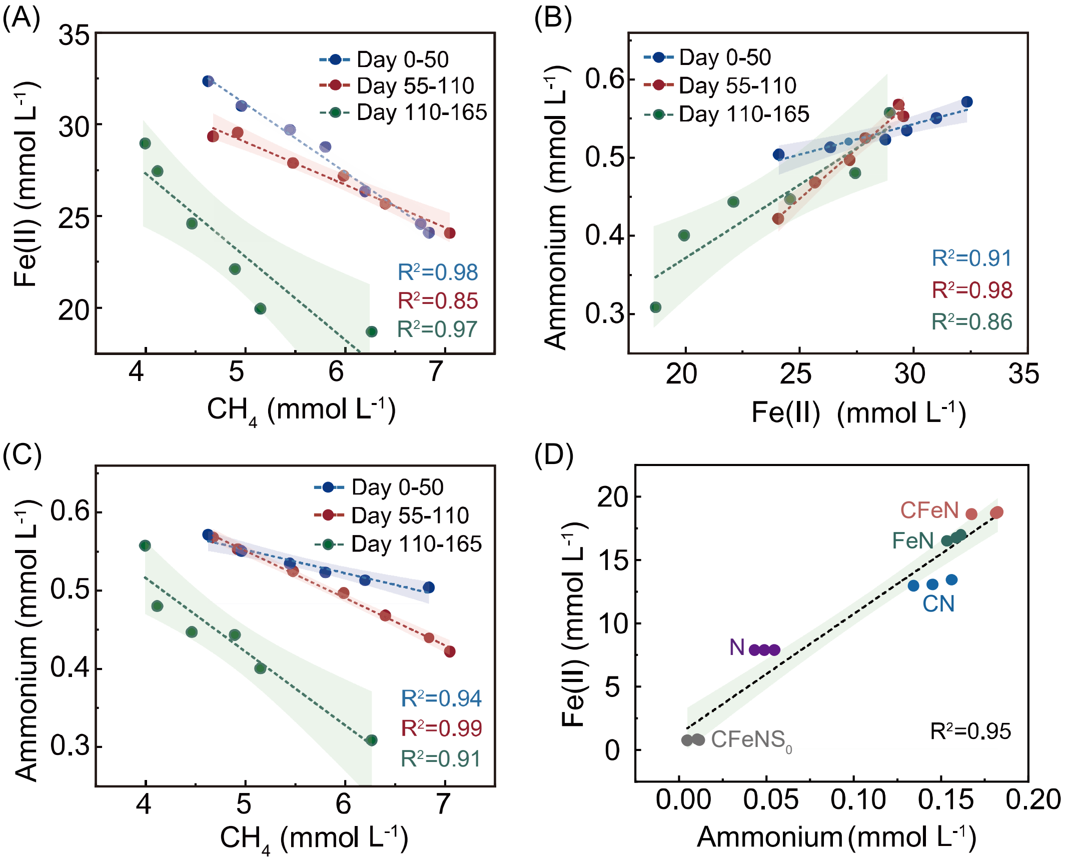

Figure S2 Linear correlations between methane consumption, Fe(II) and NH_4^+-N production for the CFeN treatment during three batch cycles (A-C) and in five different treatments (D). Data are mean ± SD of three independent biological replicates (n =3).

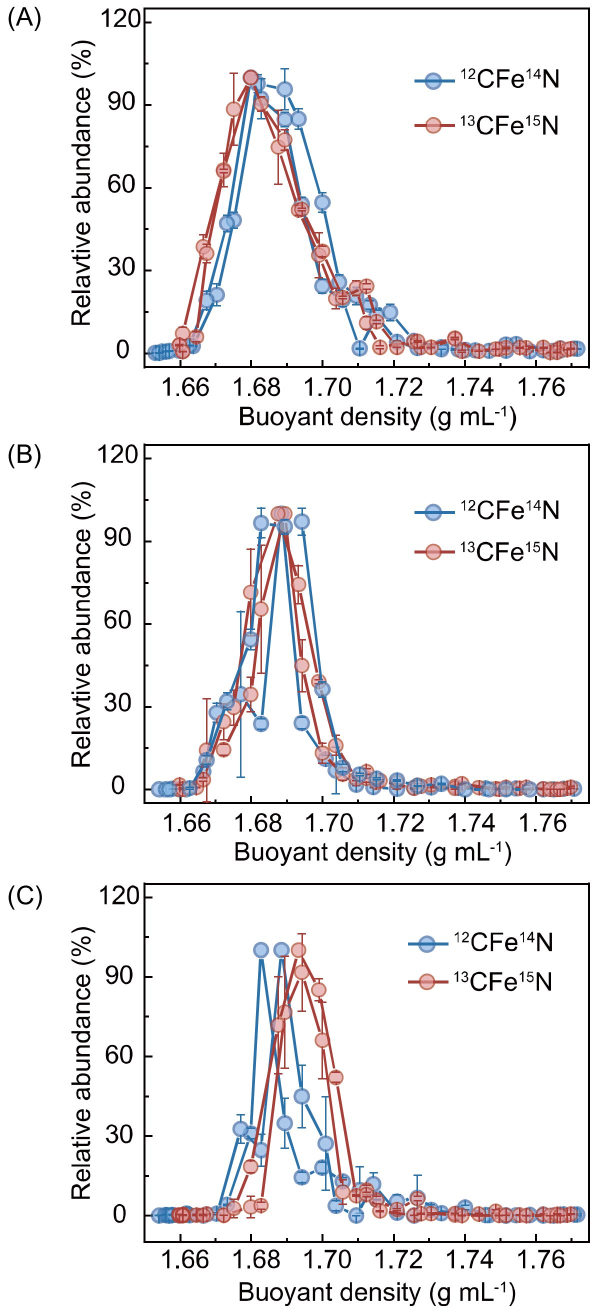

Figure S3 Normalized relative abundances of the archaeal 16S rRNA (A), *mcrA* (B) and *Geobacter* 16S rRNA genes (C) in the heavy $^{13}CFe^{15}N$-DNA fractions and light $^{12}CFe^{14}N$-DNA fractions.

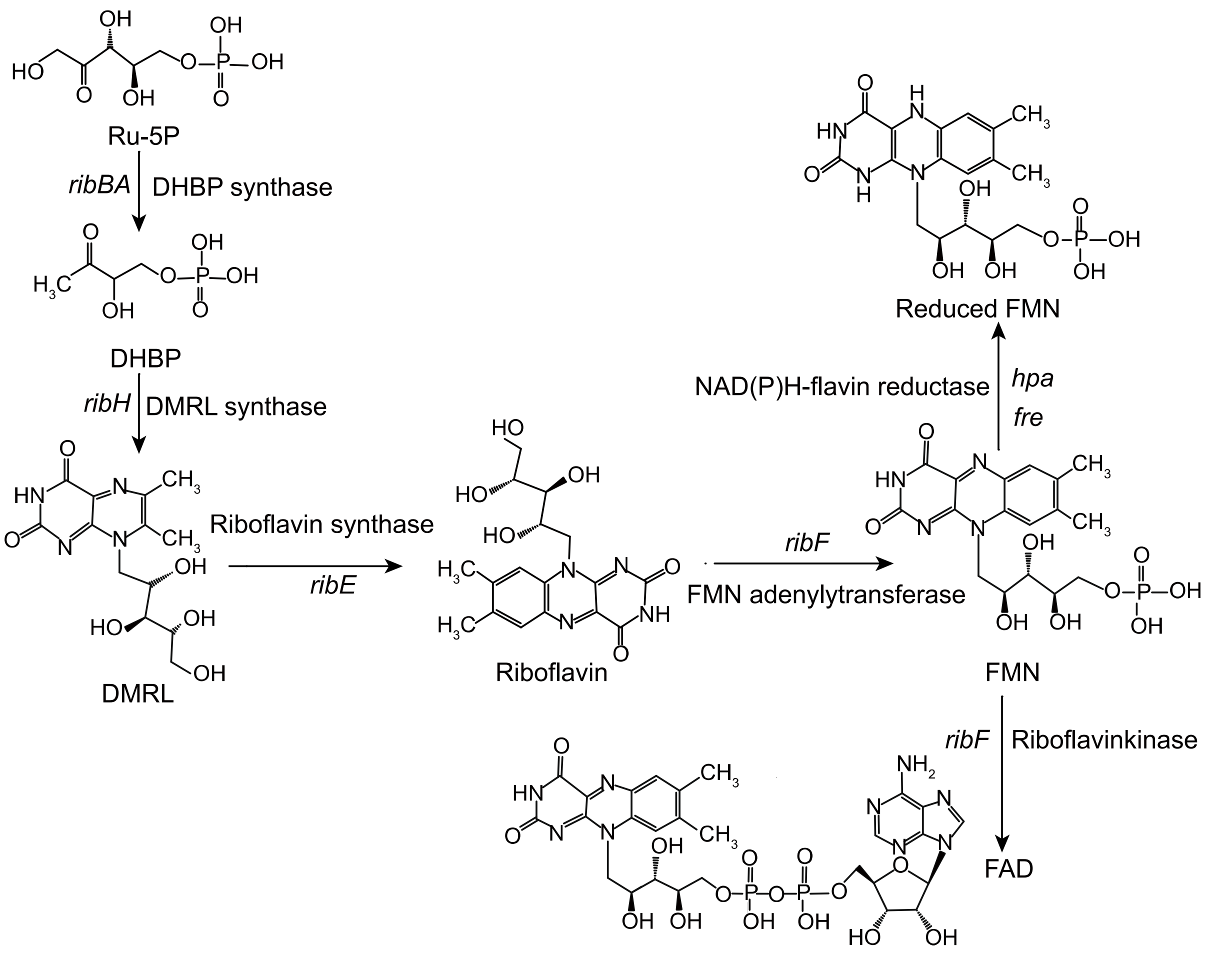

Figure S4 Predicted riboflavin biosynthesis pathway for *Methylocystis* and unclassified *Methylophilaceae* MAGs.

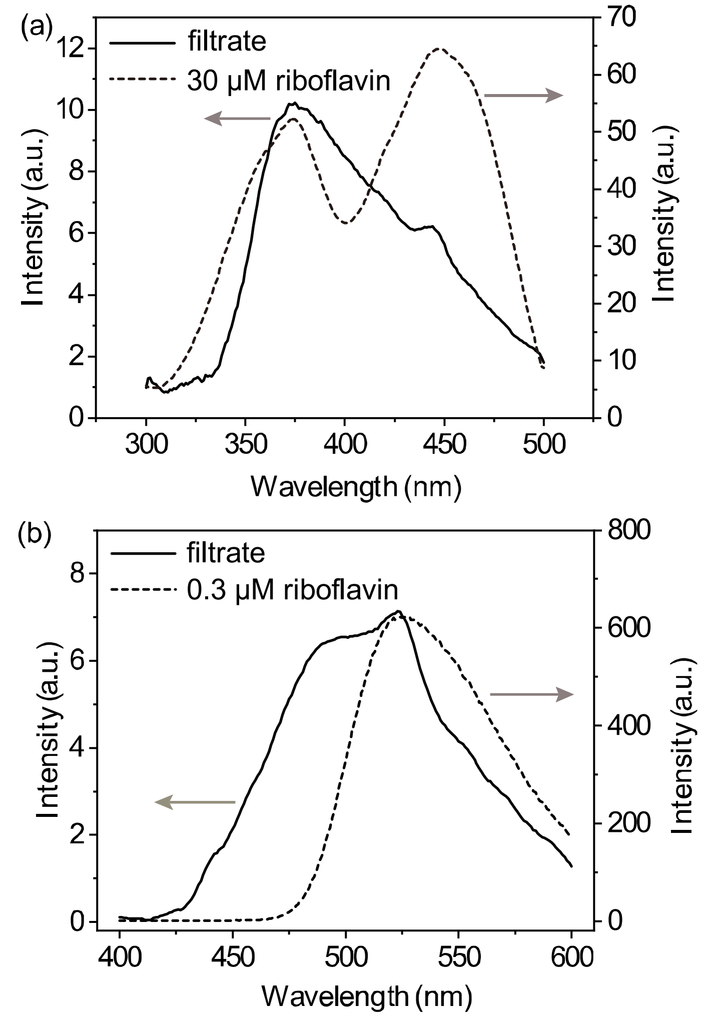

Figure S5 The presence of riboflavin in the soil filtrate revealed by fluorescence spectrometry. The excitation spectra (a) and emission spectra (b) for the filtrate and riboflavin solutions.

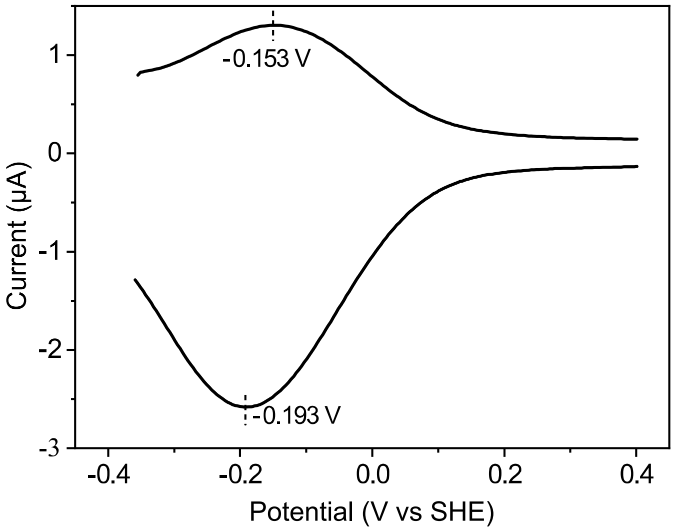

Figure S6 The DPV curves showing the oxidation and reduction peaks for the filtrate.

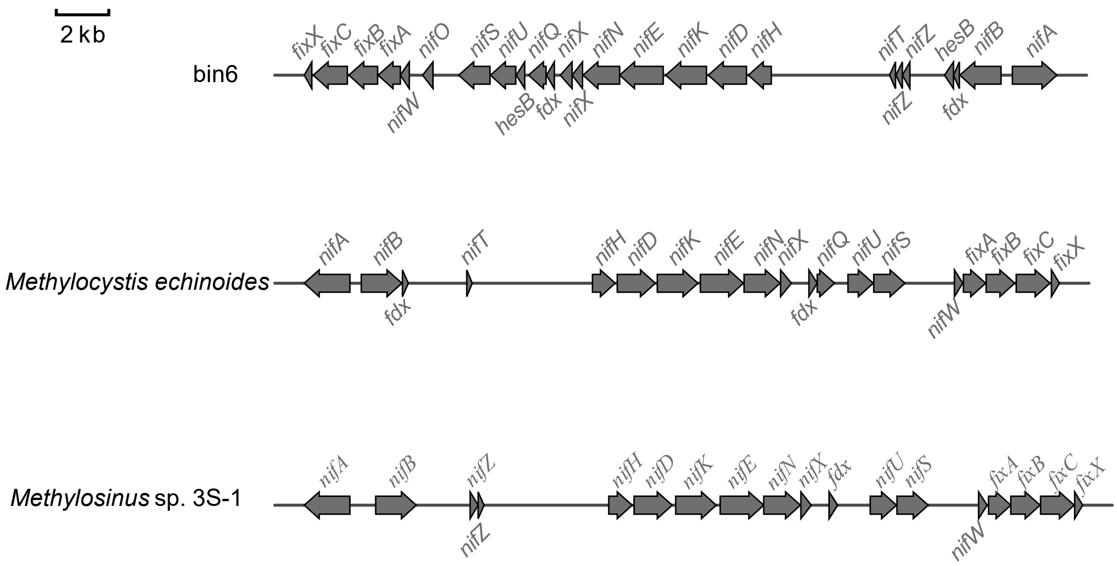

Figure S7 Organization of the *nif* and *fix* gene clusters for nitrogen fixation across different species. Arrows represent gene lengths and their respective transcription directions.

References

1. Huang L, Tang J, Chen M, Liu X, Zhou S. Two modes of riboflavin-mediated extracellular electron transfer in *Geobacter uraniireducens*. Front Microbiol. 2018;9:2886.
